# A native prokaryotic voltage-dependent calcium channel with a novel selectivity filter sequence

**DOI:** 10.7554/eLife.52828

**Published:** 2020-02-25

**Authors:** Takushi Shimomura, Yoshiki Yonekawa, Hitoshi Nagura, Michihiro Tateyama, Yoshinori Fujiyoshi, Katsumasa Irie

**Affiliations:** 1 Cellular and Structural Physiology Institute (CeSPI)Nagoya UniversityNagoyaJapan; 2 Division of Biophysics and NeurobiologyNational Institute for Physiological SciencesOkazakiJapan; 3 Graduate School of Pharmaceutical SciencesNagoya UniversityNagoyaJapan; 4 CeSPIA IncTokyoJapan; Universidad Nacional Autónoma de MéxicoMexico; The University of Texas at AustinUnited States

**Keywords:** ion selectivity, ion channels, electrophysiology, calcium channel, Other

## Abstract

Voltage-dependent Ca^2+^ channels (Cavs) are indispensable for coupling action potentials with Ca^2+^ signaling in living organisms. The structure of Cavs is similar to that of voltage-dependent Na^+^ channels (Navs). It is known that prokaryotic Navs can obtain Ca^2+^ selectivity by negative charge mutations of the selectivity filter, but native prokaryotic Cavs had not yet been identified. We report the first identification of a native prokaryotic Cav, CavMr, whose selectivity filter contains a smaller number of negatively charged residues than that of artificial prokaryotic Cavs. A relative mutant whose selectivity filter was replaced with that of CavMr exhibits high Ca^2+^ selectivity. Mutational analyses revealed that the glycine residue of the CavMr selectivity filter is a determinant for Ca^2+^ selectivity. This glycine residue is well conserved among subdomains I and III of eukaryotic Cavs. These findings provide new insight into the Ca^2+^ selectivity mechanism that is conserved from prokaryotes to eukaryotes.

## eLife digest

Electrical signals in the brain and muscles allow animals – including humans – to think, make memories and move around. Cells generate these signals by enabling charged particles known as ions to pass through the physical barrier that surrounds all cells, the cell membrane, at certain times and in certain locations.

The ions pass through pores made by various channel proteins, which generally have so-called “selectivity filters” that only allow particular types of ions to fit through. For example, the selectivity filters of a family of channels in mammals known as the Cavs only allow calcium ions to pass through. Another family of ion channels in mammals are similar in structure to the Cavs but their selectivity filters only allow sodium ions to pass through instead of calcium ions.

Ion channels are found in all living cells including in bacteria. It is thought that the Cavs and sodium-selective channels may have both evolved from Cav-like channels in an ancient lifeform that was the common ancestor of modern bacteria and animals. Previous studies in bacteria found that modifying the selectivity filters of some sodium-selective channels known as BacNavs allowed calcium ions to pass through the mutant channels instead of sodium ions. However, no Cav channels had been identified in bacteria so far, representing a missing link in the evolutionary history of ion channels.

Shimomura et al. have now found a Cav-like channel in a bacterium known as *Meiothermus ruber*. Like all proteins, ion channels are made from amino acids and comparing the selectivity filter of the *M. ruber* Cav with those of mammalian Cavs and the calcium-selective BacNav mutants from previous studies revealed one amino acid that plays a particularly important role. This amino acid is a glycine that helps select which ions may pass through the pore and is also present in the selectivity filters of many Cavs in mammals.

Together these findings suggest that the Cav channel from *M. ruber* is similar to the mammal Cav channels and may more closely resemble the Cav-like channels thought to have existed in the common ancestor of bacteria and animals. Since other channel proteins from bacteria are useful genetic tools for studies in human and other animal cells, the Cav channel from *M. ruber* has the potential to be used to stimulate calcium signaling in experiments.

## Introduction

Voltage-dependent Ca^2+^ channels (Cavs), which couple the membrane voltage with Ca^2+^ signaling, regulate some important physiological functions, such as neurotransmission and muscle contraction (Hille, 2001). The channel subunits of both mammalian Cavs and mammalian voltage-dependent Na^+^ channels (Navs) have 24 transmembrane helices (24TM) (Catterall, 2000), and comprise four homologous subdomains with six transmembrane helices that correspond to one subunit of homo-tetrameric channels, such as voltage-dependent K^+^ channels and prokaryotic Navs (BacNavs). Comparison of the sequences of Navs and Cavs indicate that Navs derived from Cavs. Their two pairs of subdomains, domains I and III, and domains II and IV, are evolutionarily close to each other (Rahman et al., 2014; Strong et al., 1993). Therefore, the 24TM-type of Cavs are thought to have evolved from the single-domain type of Cavs with two domain-duplication events. Although prokaryotes are expected to have such ancestor-like channels, native prokaryotic Cavs have not yet been identified. The lack of ancestor-like prokaryotic Cavs is a missing link in the evolution of voltage-dependent cation channels.

In contrast to the lack of prokaryotic Cavs, many BacNavs have been characterized (Irie et al., 2010; Ito et al., 2004; Koishi et al., 2004; Lee et al., 2012; Nagura et al., 2010; Payandeh et al., 2011; Ren et al., 2001; Shimomura et al., 2016; Shimomura et al., 2011). The simple structure of BacNavs has helped to elucidate the molecular mechanisms of Navs (Irie et al., 2018; Irie et al., 2012; Tsai et al., 2013; Yue et al., 2002). In addition, BacNavs have been used as a genetic tool for manipulating neuronal excitability in vivo (Bando et al., 2016; Kamiya et al., 2019; Lin et al., 2010). The acquisition of Ca^2+^ selectivity by BacNavs can be engineered by the introduction of several negatively charged amino acids into the selectivity filter (Tang et al., 2014; Yue et al., 2002). A mutant channel NavAb (a BacNav from *Arcobacter butzleri*) produced in this way, showed high Ca^2+^ selectivity, and the structural basis of Ca^2+^ selectivity has been discussed on the basis of its crystal structures (Tang et al., 2016; Tang et al., 2014). However, the selectivity filter sequences of CavAb, which were made by mutation of NavAb and contain a large number of aspartates, are quite different from those of the original mammalian Cavs. The evolutional analysis also indicated that BacNavs acquired sodium selectivity independent from that of 24TM Navs (Liebeskind et al., 2013). From these points of view, ancestor-like prokaryotic Cavs could be expected to help us to understand the structural and functional relationship between BacNavs and 24TM channels.

Here, we newly characterized two BacNav homologs, CavMr from *Meiothermus ruber* and NavPp from *Plesiocystis pacifica*. These two channels are evolutionarily distant from the previously reported canonical BacNavs. We confirmed that CavMr has clear Ca^2+^ selectivity, and that NavPp has Na^+^ selectivity with Ca^2+^-dependent inhibition. The discovery of these channels suggests the possible importance of voltage-regulated Ca^2+^ signaling in prokaryotes and may be a new genetic tool for controlling Ca^2+^ signaling. Furthermore, mutational analyses indicate that the glycine residue of the CavMr selectivity filter is important for Ca^2+^ selectivity. This glycine residue is also well conserved in the selectivity filter of subdomains I and III of mammalian Cavs. On the basis of these observations, we propose that CavMr is an ancestor-like prokaryotic Cav with a Ca^2+^ selectivity mechanism that is different from that in artificial CavAb. Further phylogenetical analyses indicated that CavMr and NavPp homologs form a wide-spread group in prokaryote and archaea, which is different from canonical BacNavs. Therefore, they are expected to advance our understanding of Ca^2+^ recognition and the evolution of voltage-dependent cation channels.

## Results

### Identification of a prokaryotic channel with Ca^2+^ permeability

**Figure 1.**
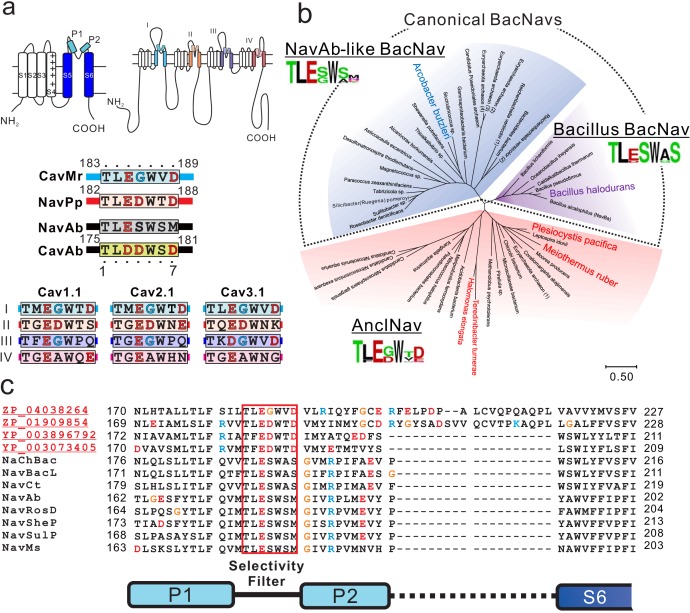
Sequence analysis of ancestor-like BacNavs. (**a**) Schematic secondary structure and selectivity filter sequence of BacNavs and 24TM channels. A cylinder indicates an α-helix. The selectivity filter sequences are indicated using single-letter codes. Negatively charged residues are colored in red. Glycine residues in the position four are colored in cyan. The straight lines indicate the other parts of the pore domain. The selectivity filter sequences of hCav1.1 (UniProt ID: Q13698), hCav2.1 (O00555) and hCav3.1 (O43497) were used. (**b**) Phylogenetic tree of canonical BacNavs and ancestor-like BacNavs (AnclNavs). The MUSCLE program was used to align the multiple protein sequences of the channels (Figure 1—source data 1). The phylogenetic tree was generated using MEGA X. The branch lengths are proportional to the sequence divergence, with the scale bar corresponding to 0.5 substitutions per amino acid position. Three phylogenetically distinct groups are shown in different background colors (purple, Bacillus BacNavs; blue, NavAb-like BacNavs; red, AnclNavs). Four homologs with the taxon name colored in red in the AnclNav group were cloned and expressed to check the channel activity. Two of those, which are shown in larger and bold text, generated the detectable currents. The appearance frequency of amino acids in each of the selectivity filter sequences is shown under the respective group names. (**c**) Alignment of the deduced amino-acid sequences of the P1 helix to P2 helix domain of novel cloned homologs of AnclNavs with well characterized BacNavs. Figure 1—source data 1.Amino-acid sequences used for making phylogenetic tree.

We searched for the primary sequences of candidate prokaryotic Cavs in the GenBank database. In mammalian and prokaryotic Navs and Cavs, a larger number of negative charges in the filter increases Ca^2+^ selectivity (Figure 1a) (Heinemann et al., 1992; Tang et al., 2014; Yue et al., 2002). Several BLAST search rounds using the pore regions (S5–S6) of NaChBac (or NavBh; a BacNav from *Bacillus halodurans*) as templates revealed a series of candidate prokaryotic Cavs whose selectivity filters are similar to the ‘TLESW’ motif, but which contain more negatively charged residues like the filter sequence of CavAb (Figure 1a). Phylogenetic analysis of these channel genes revealed that they apparently belong to a different branch of the tree than that of canonical BacNavs, namely a *Bacillus* group and a NavAb-like group (Figure 1b; Figure 1—source data 1). The selectivity filter sequences of these channels are similar to that of the ancestral BacNav channel predicted previously (Liebeskind et al., 2013). Therefore, we named these channels ancestor-like BacNavs (AnclNavs).

**Figure 1—figure supplement 1.**
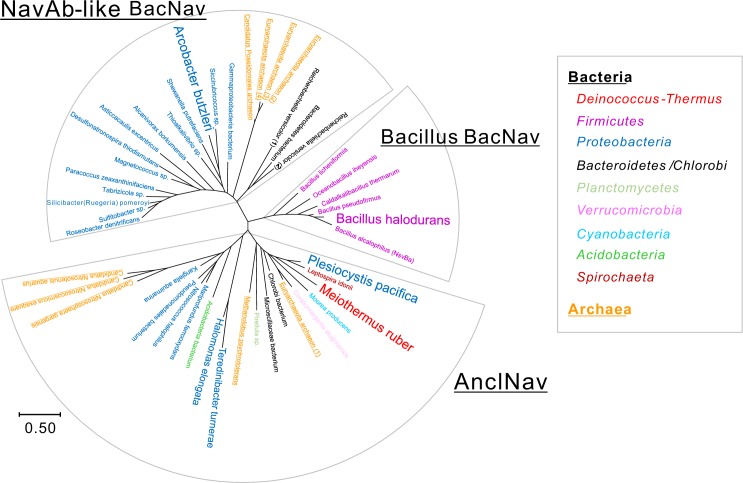
Distribution of the bacterial phylum and archaea in the phylogenetic tree of BacNavs. Bacterial species that have a BacNav homolog are separately colored according to their different phyla, plus the archaea colored in orange. The branch lengths are proportional to the sequence divergence, with the scale bar corresponding to 0.5 substitutions per amino acid position.

**Figure 1—figure supplement 2.**
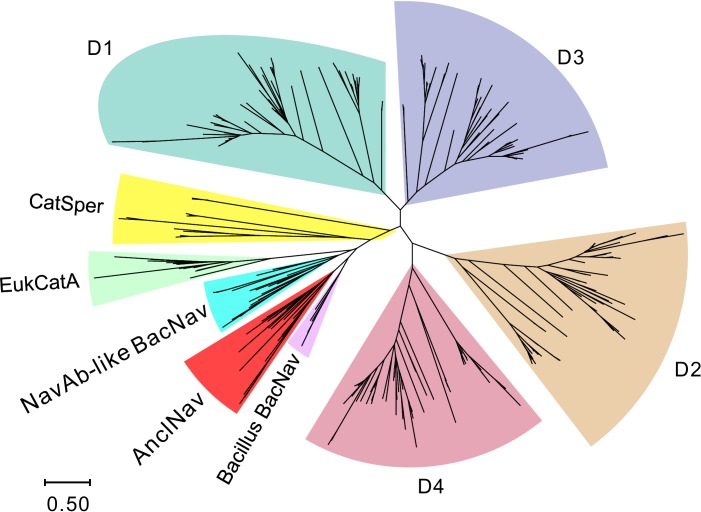
Phylogenetic analysis of BacNavs with eukaryotic voltage-gated cation channels. Protein sequences of eukaryotic Cavs and Navs (Figure 1—figure supplement 2—source data 1) were divided into four subdomains and phylogenetically analyzed (D1–D4) with other single-domain channel types, including CatSper (yellow) and diatom EukCatA (dark green) channels. The branch lengths are proportional to the sequence divergence, with the scale bar corresponding to 0.5 substitutions per amino acid position. Figure 1—figure supplement 2—source data 1.Amino-acid sequences used for making phylogenetic tree.

AnclNavs are widely distributed in multiple bacterial phyla and even in archaea (Figure 1—figure supplement 1; Figure 1—source data 1). In some cases, one phylum, such as proteobacteria, contains both a NavAb-like BacNav and an AnclNav gene. Even in those cases, the NavAb-like BacNav and the AnclNav were included separately in their respective groups. The bacillus BacNav group is a different group located phylogenetically between the NavAb-like and the AnclNav groups. In addition, the firmicutes phylum, which includes Bacillus species, contains neither homologs of NavAb-like BacNavs nor homologs of AnclNavs. These observations suggest that our identified candidate prokaryotic Cavs, AnclNavs, are homologs rather than orthologs of canonical BacNavs and compose a distinct group. In addition, analyses that include some eukaryotic channels, such as each subdomain of 24TM-type of Navs/Cavs, CatSper and EukCatA, a group of eukaryotic non-selective homotetrameric channels, put AnclNavs closest to a *Bacillus* group (Figure 1—figure supplement 2; Figure 1—figure supplement 2—source data 1).

**Figure 2.**
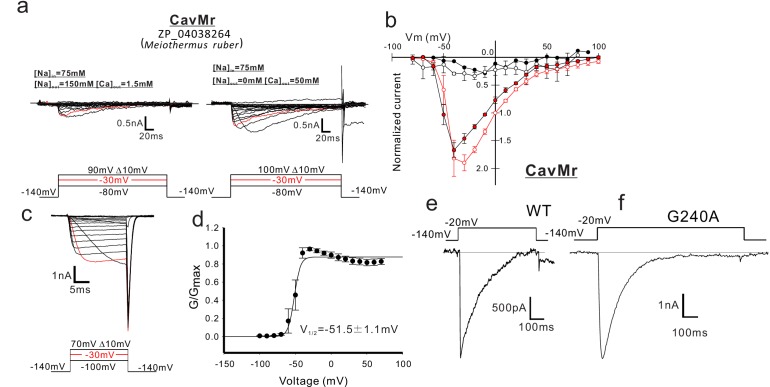
Functional expression of CavMr in SF-9 insect cells. (**a**) Representative current traces used to describe the current-voltage relationships of CavMr in SF9 cells. The horizontal lines are superimposed to indicate the zero-current level in the representative current traces. Currents were generated in the bath solutions containing high Na^+^ (left) and high Ca^2+^ (right), by a series of step-pulses (shown at the bottom of the panel). (**b**) Current-voltage relationships of CavMr measured in the different bath solutions [filled black, 150 mM NaCl (*n* = 4); open black, 75 mM NaCl and 75 mM NMDG-HCl (*n* = 4); open red, 75 mM NaCl and 50 mM CaCl_2_ (*n* = 7); filled red, 50 mM CaCl_2_ and 75 mM NMDG-HCl (*n* = 6)] (Figure 2—source data 1). Currents of CavMr were normalized to that invoked by 0 mV depolarization stimuli under 75 mM NaCl and 50 mM CaCl_2_ bath solution. (**c**) Deactivation tail currents of CavMr. After prepulses of varying depolarization (bottom), tail currents were measured at −140 mV. (**d**) G/G_max_ curve of CavMr generated by tail currents (*n* = 6) (Figure 2—source data 2). (**e, f**) Whole-cell currents in CavMr wild type [WT; (e) ] and a G240A mutant (**f**) when a pulse of −20 mV was given for 500 ms and 1 s, respectively, in a high Ca^2+^ bath solution. Figure 2—source data 1.The values of the currents generated by each voltage stimulation. Figure 2—source data 2.The values of G/Gmax of CavMr derived from the tail currents generated by each voltage stimulation.

We identified four AnclNavs genes and measured their channel activity: ZP_04038264 from *M. ruber*, ZP_01909854 from *P. pacifica*, YP 003896792_from *Halomonas elongata*, and YP_003073405 from *Teredinibacter turnerae* (Figure 1b and c). When attempting to express prokaryotic channels transgenetically, insect cells are often better than mammalian cells for generating large current amplitudes (Irie et al., 2018). We therefore transfected Sf9 cells with these four channel genes and measured the resulting whole-cell currents. The cells that were transfected with genes from *M. ruber* showed currents in response to a depolarizing stimulus from a −140 mV holding potential (Figure 2a). To estimate the Ca^2+^ permeability, we measured their current-voltage relationships. The *M. ruber* channel clearly had larger currents in the high-Ca^2+^ solution than in the high-Na^+^ solution, and no obvious outward current was observed in a high-Ca^2+^ bath solution, even at very high membrane potential (100 mV) (Figure 2a and b; Figure 2—source data 1). These current-voltage relationships suggest that the *M. ruber* channel has a preference for Ca^2+^, and that other cations inside the cells (sodium and cesium) hardly permeated the activated channel. Therefore, the newly identified channel from *M. ruber* is abbreviated as CavMr, based on its ion selectivity and species name. We evaluated the voltage-dependent activation of CavMr by measuring deactivation tail currents (Figure 2c). A Boltzmann fit of the averaged activation curve yielded an activating potential of 50% activation (V_1/2_) of −51.7 ± 1.1 mV (Figure 2d; Figure 2—source data 2).

To compare clearly the positions of the residues in the selectivity filter in each channel, we renumbered the seven residues comprising the selectivity filter. For example, the seven residues of the CavMr selectivity filter are 183-TLEGWVD-189, and thus Thr183 and Asp189 were renumbered as Thr1 and Asp7 (Figure 1a). Notably, the amino acid sequence of the selectivity filter in CavMr is similar to the conserved features of domains I/III in mammalian Cavs, with a glycine at position 4 and a polar or negatively charged residue at position 7, which are not observed in the canonical BacNav family. In addition, the CavMr selectivity filter sequence is quite similar to that of the human Cav subdomain I, or even the same as Cav3.1 and 3.2 (Figure 1a).

In the following experiments, to evaluate the reversal potential for the ion selectivity analysis accurately, we introduced a single mutation that resulted in large and long-lasting channel currents. T220A and G229A mutations in NaChBac led to slower inactivation and provided a larger current, indicating suppression of the transition to the inactivated state (Irie et al., 2010; Shimomura et al., 2016). We introduced a G240A mutation to CavMr, corresponding to the NaChBac mutations of G229A. Th is mutant channels stably showed larger and more measurable currents than the wild-type channel, even after they were administrated multiple depolarizing stimuli (Figure 2e and f).

### CavMr has high Ca^2+^ selectivity over Na^+^

**Figure 3.**
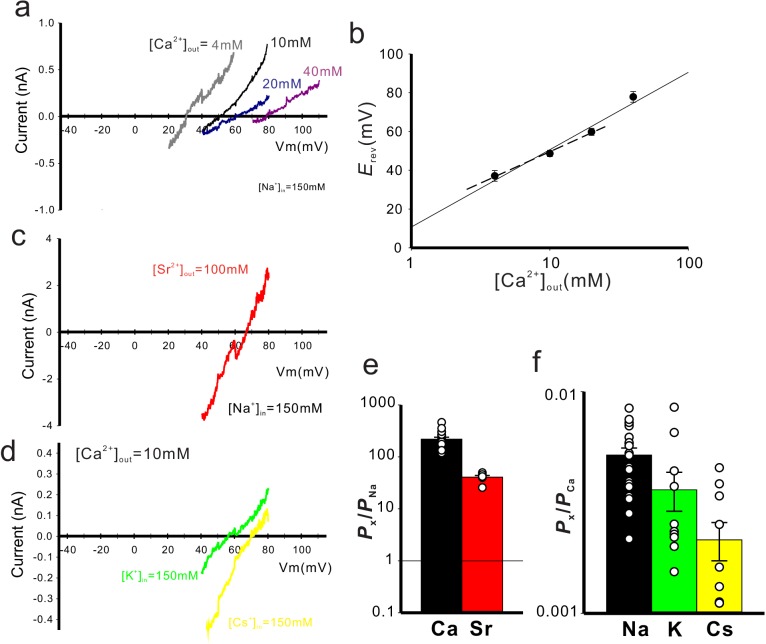
Cation selectivity of CavMr. (**a**) Current-voltage relationship plot generated by ramp pulses in various [Ca^2+^]_out_ and 150 mM [Na^+^]_in_. (**b**) The plot of the reversal potential to [Ca^2+^]_out_. Each value was obtained using the ramp pulse protocol shown in panel (a) (Figure 3—source data 1). The relationship was fitted by a line with the slope of 39.89 ± 3.31 mV per decade (*n* = 7). (**c**) Current-voltage relationship plot generated by ramp pulses in 100 mM [Sr^2+^]_out_ and 150 mM [Na^+^]_in_. (**d**) Current-voltage relationship plots generated by ramp pulses in 10 mM [Ca^2+^]_out_ and 150 mM [K^+^]_in_ or [Cs^+^]_in_. (**e**) The relative permeability of Ca^2+^ or Sr^2+^ to Na^+^ in CavMr, calculated from the reversal potentials that were obtained by the ramp pulses shown in Figure 3—figure supplement 1 (Figure 3—source data 2). (**f**) The relative permeability of each monovalent cation to Ca^2+^ in CavMr, derived from the data shown in Figure 3—figure supplement 1 (Figure 3—source data 3). Figure 3—source data 1.The reversal potentials to each extracellular Ca^2+^concentration. Figure 3—source data 2.The values of relative permeability. Figure 3—source data 3.The values of relative permeability.

**Figure 4.**
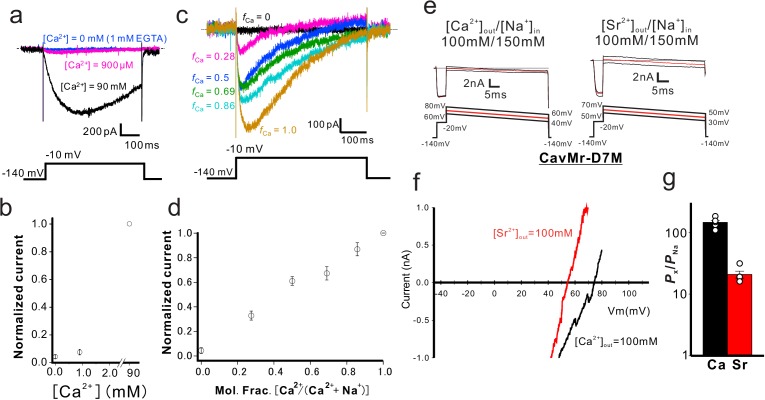
Characterization of the selectivity filter of CavMr. (**a**) Examination of anomalous mole fraction effects in CavMr. CavMr currents were recorded in a bath solution containing the following ratios of Na^+^ and Ca^2+^ ([Na^+^]:[Ca^2+^]) — 0:90, 133.7:0.9 and 135:0 (mM), respectively. The 0 mM Ca^2+^ solution also contains 1 mM EGTA. (**b**) Plot of the normalized current amplitude of CavMr obtained from panel (a) (*n* = 3) (Figure 4—source data 1). (**c**) Representative current traces of CavMr under different mole fractions of Ca^2+^. *f*_Ca_ indicates [Ca^2+^]_out_ / ([Ca^2+^]_out_ + [Na^+^]_out_). (**d**) Plot of the normalized current amplitude to each mole fraction, as measured in c (*n* = 5) (Figure 4—source data 2). (**e**) For the evaluation of the relative permeability of Ca^2+^ and Sr^2+^ to Na^+^ of CavMr-D7M, Ca^2+^ solution [100 mM CaCl_2_, 10 mM HEPES (pH 7.4 adjusted with Ca(OH)_2_) and 10 mM glucose] and Sr^2+^ solution [100 mM SrCl_2_, 10 mM HEPES (pH 7.4 adjusted by Sr[OH]_2_) and 10 mM glucose] were used as bath solutions. High-Na^+^ pipette solution [115 mM NaF, 35 mM NaCl, 10 mM EGTA, and 10 mM HEPES (pH 7.4 adjusted by NaOH)] was used. Currents were generated by the step pulse of −20 mV from −140 mV holding potential, followed by ramp pulses with different voltage values. The time courses of the change of membrane potentials are shown at the bottom of each current traces. (**f**) Current-voltage relationship plots generated by ramp pulses in 150 mM [Na^+^]_in_ and 100 mM [Ca^2+^]_out_ or [Sr^2+^]_out_. (**g**) The relative permeability of divalent cations to Na^+^ in CavMr-D7M, whose position 7 residue in the selectivity filter was neutralized by the corresponding residue of NavAb (Figure 4—source data 3). Figure 4—source data 1.The values of the normalized current amplitude of CavMr. Figure 4—source data 2.The values of the normalized current amplitude to each mole fraction. Figure 4—source data 3.The values of relative permeability.

We accurately quantified the selectivity of CavMr for Na^+^ and Ca^2+^ (*P*_Ca_/*P*_Na_) by measuring the reversal potential (E_rev_) under bi-ionic conditions, in which the Ca^2+^ concentration in the bath solution was changed to 4, 10, 20, and 40 mM while the intracellular Na^+^ concentration was held constant at 150 mM (Figure 3a and b; Figure 3—figure supplement 1a–d; Figure 3—source data 1). The plot of the reversal potentials as a function of [Ca^2+^]_out_ had a slope of 39.89 ± 3.31 mV/decade (*n* = 4). It was higher than the Nernst prediction for a divalent cation (29 mV). We think that this deviation came from incorporation of the measurements made with the 40 mM Ca^2+^ bath solution, in which no obvious outward current was observed (Figure 3—figure supplement 1d), resulting in a higher value of *E*_rev_. The well-fitted plot of the reversal potentials in 4, 10 and 20 mM Ca^2+^ bath solutions as a function of [Ca^2+^]_out_did indeed have a slope of 32.38 ± 4.10 mV/decade (*n* = 4) (Figure 3b dashed line), a value close to the Nernst equation for Ca^2+^ (Figure 3b), and indicated that CavMr had a *P*_Ca_/*P*_Na_ of 218 ± 38 (Figure 3e, Table 1; Figure 3—source data 2). This high *P*_Ca_/*P*_Na_ value is comparable to that of CavAb. Among several species of cations, including Sr^2+^, K^+^, and Cs^+^, Ca^2+^ had the highest permeability relative to Na^+^ (Figure 3c,d and f, Table 1; Figure 3—source data 3). On the basis of these results, CavMr was confirmed to be a native prokaryotic Cav with high Ca^2+^ selectivity. We also investigated whether CavMr shows the typical anomalous mole fraction effect (Almers and McCleskey, 1984) and the non-monotonic mole fraction effect observed in NaChBac (Finol-Urdaneta et al., 2014). CavMr did not allow Na^+^ permeation under Ca^2+^-free (0 mM CaCl_2_ and 1 mM EGTA) conditions (Figure 4a and b; Figure 4—source data 1). Also, in contrast to the recording of NaChBac currents in a solution containing Na^+^ and K^+^, CavMr had an apparently monotonic current increase depending on the Ca^2+^ mole fraction to Na^+^ (Figure 4c and d; Figure 4—source data 2).

**Figure 3—figure supplement 1.**
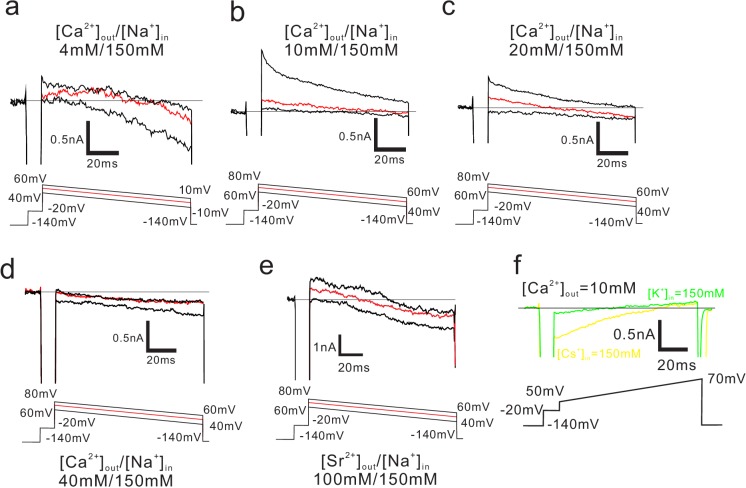
Representative current traces of CavMr generated by the ramp protocol. (**a–d**) Recordings of the reversal potential of CavMr currents obtained using the ramp protocol. Currents were generated by the step pulse of −20 mV from −140 mV holding potential, followed by the ramp pulses with different voltage values (shown at the bottom). The values of the reversal potential recorded with three different ramp pulses were averaged and used in Figure 3. Currents were measured in the bath solution containing 4 mM (**a**), 10 mM (**b**), 20 mM (**c**) and 40 mM (**d**) CaCl_2_ and with a pipette solution containing 150 mM [Na^+^]. (**e**) Representative current traces of ramp pulses used to obtain the reversal potential under conditions of 100 mM [Sr^2+^]_out_ and 150 mM [Na^+^]_in_. Currents were generated using the protocol shown in the lower part. (**f**) Representative current traces investigating the *P*_Cs_/*P*_Ca_ and *P*_K_/*P*_Ca_, the pipette solutions contained 150 mM [Cs^+^] for *P*_Cs_/*P*_Ca_ and 150 mM [K^+^] for *P*_K_/*P*_Ca_, while the bath solution contained 10 mM [Ca^2+^] in both cases.

**Table 1.** Relative permeability of CavMr and NavPp. All values are indicated as mean ± S.E.

	*P*_Ca_/*P*_Na_	_Sr_/*P*_Na_	*P*_K_/*P*_Na_	*P*_Cs_/*P*_Na_
CavMr G240A	218 ± 38	40.6 ± 3.4	0.0036 ± 0.00072^a^	0.0021 ± 0.00042^b^
	(*n *= 20)	(*n *= 6)	(*n *= 10)	(*n *= 10)
Pp	13.8 ± 2.0	24.5 ± 0.3	0.95 ± 0.04	0.57 ± 0.05
	(*n *= 7)	(*n *= 5)	(*n *= 4)	(*n *= 3)
G4D	7.73 ± 2.24	18.6 ± 6.1	1.20 ± 0.28	0.87 ± 0.21
	(*n *= 11)	(*n *= 4)	(*n *= 4)	(*n *= 4)
G4S	11.9 ± 1.5	4.23 ± 0.27	1.54 ± 0.12	2.02 ± 0.48
	(*n *= 5)	(*n *= 5)	(*n *= 5)	(*n *= 3)
V6T	40.1 ± 9.7	13.3 ± 2.5	0.69 ± 0.26	0.54 ± 0.60
	(*n *= 5)	(*n *= 5)	(*n *= 3)	(*n *= 3)
D7M	144 ± 12	20.7 ± 2.7	N.D.	N.D.
	(*n *= 5)	(*n *= 5)		
NavPp T232A	0.308 ± 0.028	0.38 ± 0.027	0.16 ± 0.026	0.0052 ± 0.0006
	(*n *= 18)	(*n *= 9)	(*n *= 9)	(*n *= 7)
Mr	215 ± 33	86.3 ± 12.2	0.0045 ± 0.00072^a^	0.0135 ± 0.0039^b^
	(*n *= 7)	(*n *= 4)	(*n *= 4)	(*n *= 8)
D4G	41.4 ± 6.7	8.85 ± 0.95	0.81 ± 0.11	0.56 ± 0.05
	(*n *= 10)	(*n *= 4)	(*n *= 3)	(*n *= 4)
T6V	1.72 ± 0.19	33.9 ± 5.0	0.99 ± 0.03	0.84 ± 0.02
	(*n *= 10)	(*n *= 8)	(*n *= 4)	(*n *= 4)

^a^ Because of high Ca^2+^ selectivity, *P*_K_/*P*_Ca_ are indicated. ^b^ Because of high Ca^2+^ selectivity, *P*_Cs_/*P*_Ca_ are indicated.

Studies of an artificial Cav, CavAb, revealed that Ca^2+^ selectivity depends on the presence of a large number of aspartates in the filter sequence (Tang et al., 2014). The high Ca^2+^ selectivity in CavMr was unexpected because the filter sequence contained only one aspartate residue (Figure 1c). Furthermore, CavMr-D7M, which has only one negatively charged residue in the selectivity filter ‘TLEGWVM’, still had high Ca^2+^ selectivity, comparable to that of wild-type CavMr (*P*_Ca_/*P*_Na_ = 144 ± 12; Figure 4e-g and Table 1; Figure 4—source data 3). These findings indicate that CavMr and artificial CavAb have different Ca^2+^-selection mechanisms.

### NavPp is permeable to Na^+^ and is blocked by extracellular Ca^2+^

**Figure 5.**
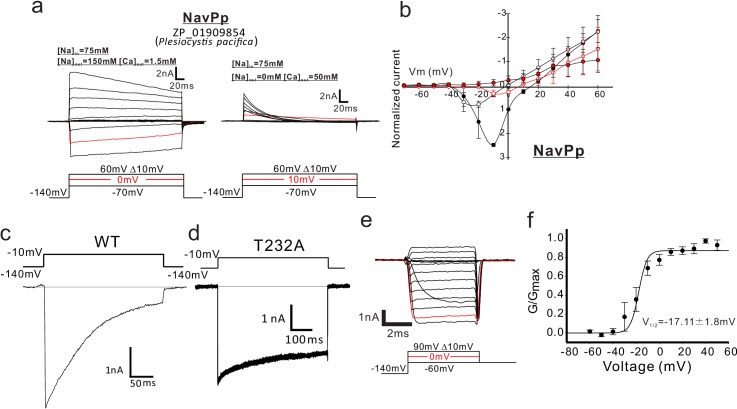
Functional expression of NavPp in SF-9 insect cells. (**a**) Representative current traces used to obtain the current-voltage relationships of NavPp in SF9 cells. The horizontal lines are superimposed to indicate the zero-current level in the representative current traces. Currents were generated, in bath solutions containing high Na^+^ (left) and high Ca^2+^ (right), by a series of step-pulses shown at the bottom of the panel. (**b**) Current-voltage relationships of NavPp measured in the different bath solutions [filled black, 150 mM NaCl (*n* = 8), open black, 75 mM NaCl and 75 mM NMDG-HCl (*n* = 8); open red, 75 mM NaCl and 50 mM CaCl_2_ (*n* = 6); filled red, 50 mM CaCl_2_ and 75 mM NMDG-HCl (*n* = 8)] (Figure 5—source data 1). Currents of NavPp were normalized to that induced by 0 mV depolarization stimuli in a 150 mM NaCl bath solution. (**c,** **d**) Whole-cell recordings of wild-type NavPp [WT; (c)] and the NavPp T232A mutant (**d**) when a pulse of −10 mV was given for 250 ms and 500 ms in a high-Na^+^ bath solution, respectively. (**e**) Deactivation tail currents of NavPp T232A. After prepulses of varying depolarizing currents (bottom), tail currents were measured at −60 mV. (**f**) G/G_max_ curve for NavPp T232A derived from the tail currents (*n* = 4) (Figure 5—source data 2). Figure 5—source data 1.The values of the currents generated by each voltage stimulation. Figure 5—source data 2.The values of G/G_max_of NavPp T232A derived from the tail currents generated by each voltage stimulation.

The currents derived from the *P. pacifica* channel became large with increases in the bath Na^+^ concentration and significantly decreased when the Na^+^ solution was replaced with a high Ca^2+^ solution (Figure 5a and b; Figure 5—source data 1). Because the reversal potential fit well to the Na^+^ equilibrium potential in the high-Na^+^ solution (Figure 5b), we abbreviated this channel as NavPp on the basis of its ion selectivity and species name. Interestingly, NavPp, despite having one more aspartate in the selectivity filter than CavMr, exhibited larger currents in Na^+^ solutions than in Ca^2+^ solutions (Figure 1c and Figure 5a). This observation indicates that Ca^2+^ selection is not achieved simply by increasing negative charges in the filter sequence of the AnclNav group, which again suggests the existence of an alternative ion-selectivity mechanism.

**Figure 6.**
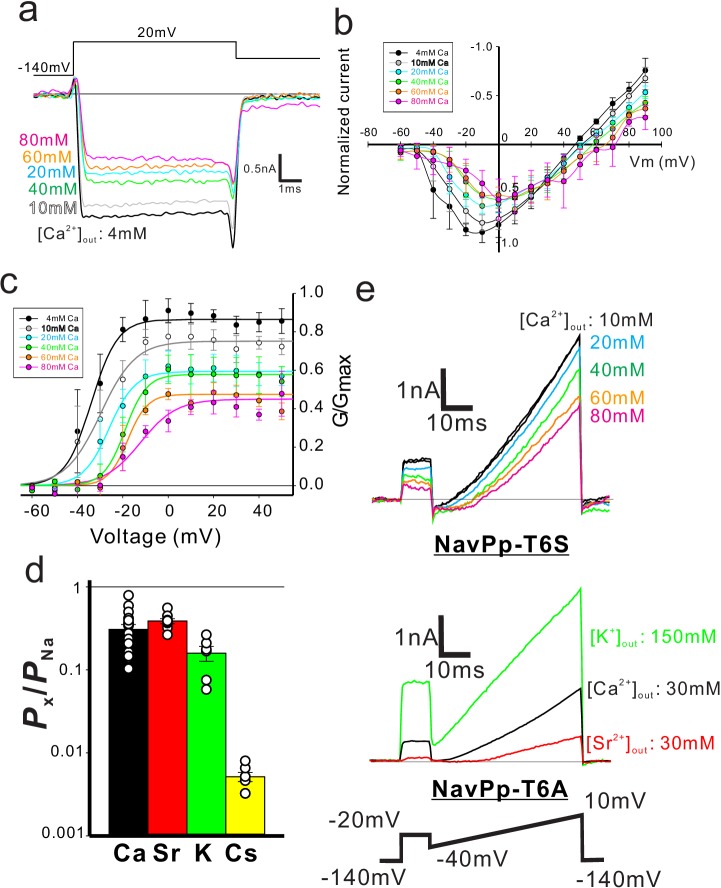
Characterization of the selectivity filter of NavPp. (**a**) Representative current traces for NavPp T232A generated by +20 mV stimulation pulses in various extracellular Ca^2+^ concentration solutions. (**b**) Current-voltage relationships of NavPp measured in various extracellular Ca^2+^ concentration solutions (*n* = 3) (Figure 6—source data 1). All values were normalized by the peak current amplitude in the 4 mM extracellular Ca^2+^ condition. (**c**) G/G_max_ curve for NavPp T232A derived from the tail currents in various extracellular Ca^2+^ concentration solutions (*n* = 4) (Figure 6—source data 2). The maximum tail current amplitude in the 4 mM extracellular calcium condition was used as G_max_. (**d**) The permeability of different cation species relative to Na^+ ^permeability in NavPp, calculated from the reversal potential that was obtained from the current traces of Figure 6—figure supplement 1b–e (Figure 6—source data 3). (**e**) The extracellular-calcium-inhibition in the single-point mutants of NavPp. The selectivity filter of NavPp was changed to the Ca^2+^-selective canonical-BacNavs mutants (T6S; TLEDWSD and T6A; TLEDWAD). Figure 6—source data 1.The values of the currents generated by each voltage stimulation under each extracellular Ca^2+^concentration. Figure 6—source data 2.The values of G/Gmax of NavPp T232A derived from the tail currents generated by each voltage stimulation under each extracellular Ca^2+^concentration. Figure 6—source data 3.The values of relative permeability.

Recordings in bath solution containing both Na^+^ and Ca^2+^ demonstrated that the increment of the extracellular Ca^2+^ decreased the current in NavPp and led to a positive shift in the voltage dependence, suggesting that a higher concentration of Ca^2+^ inhibited the gating and ionic permeation of NavPp (Figure 5b). We tried to measure the voltage-dependent activation of NavPp, but the wild-type channel showed very fast deactivation and no tail current was observed (Figure 5c). By introducing a T232A mutation, corresponding to the NaChBac mutations of T220A (Shimomura et al., 2016), we were able to observe the tail current at −60 mV (Figure 5d and e). A Boltzmann fit of the averaged activation curve of NavPp T232A yielded a V_1/2_ of −17.11 ± 1.8 mV (Figure 5f; Figure 5—source data 2). To characterize the effect of extracellular Ca^2+^ on the NavPp channel, the voltage dependence of activation of NavPp was measured under various bath Ca^2+^ concentrations (Figure 6a–c and Figure 6—figure supplement 1a; Figure 6—source data 1 and Figure 6—source data 2). The increments of the extracellular Ca^2+^ raised the value of the reversal potential, indicating that extracellular calcium ions can also permeate NavPp (Figure 6b). However, in higher extracellular Ca^2+^ concentrations, the current amplitude of NavPp became smaller, even in the voltage at which NavPp opens fully, and the voltage dependence of the activation shifted more positively (Figure 6b–c). These results indicated that calcium ions can permeate NavPp but disturb the gating and ionic permeation of NavPp.

**Figure 6—figure supplement 1.**
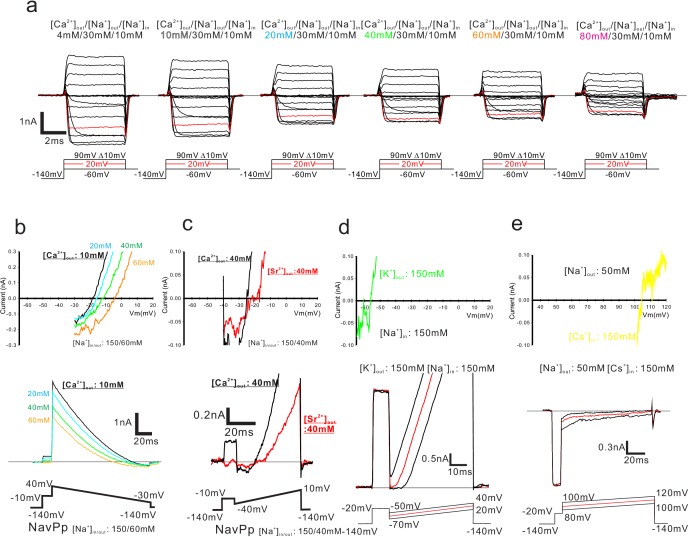
Representative current traces of NavPp generated by the step-up pulses and the ramp protocol. (**a**) Representative current traces used to obtain the peak current and the deactivation tail current for evaluation of the current-voltage relationship and the voltage-dependent activation of NavPp T232A. (**b**) Current-voltage relationship plots (upper) and representative currents (bottom) used to obtain the reversal potential in NavPp for *P*_Ca_/*P*_Na_. Currents were generated by the ramp protocol shown at the bottom of the panel. [Ca^2+^]_out_ was varied from 10 mM to 60 mM with the [Na^+^] fixed in both the bath (60 mM) and pipette (150 mM). (**c**) Current-voltage relationship plots (upper) and representative currents (bottom) used to obtain the reversal potential in NavPp for *P*_Sr_/*P*_Na_. Currents were generated by the ramp protocol shown at the bottom of the panel. [Sr^2+^]_out_ was 40 mM with the[Na^+^] fixed in both the bath (40 mM) and pipette (150 mM). (**d, e**) Current-voltage relationship plots (upper) and representative currents (bottom) used to evaluate the permeability of K^+^ and Cs^+ ^relative to that of Na^+^ of NavPp. K^+^ solution [150 mM KCl, 2 mM CaCl_2_, 10 mM HEPES (pH 7.4 adjusted by KOH) and 10 mM glucose] and low Na^+^ solution [50 mM NaCl, 100 mM NMDG-HCl, 2 mM CaCl_2_, 10 mM HEPES (pH 7.4 adjusted by NaOH) and 10 mM glucose] were used as bath solution. High-Na^+^ pipette solution [115 mM NaF, 35 mM NaCl, 10 mM EGTA, and 10 mM HEPES (pH 7.4 adjusted by NaOH)] and Cs^+^ pipette solution [115 mM CsF, 35 mM CsCl, 10 mM EGTA, and 10 mM HEPES (pH 7.4 adjusted by CsOH)] were used for K^+^ and Cs^+^ selectivity, respectively. Currents were generated by the step pulse of −20 mV from −140 mV holding potential, followed by the ramp pulses with different voltage values. The time courses of the change of membrane potentials were shown at the bottom of the respective current traces.

We then compared the relative permeability of various cations with that of Na^+^ in NavPp. The reversal potential was obtained under an extracellular solution containing Na^+^ ions, despite a partial Ca^2+^- or Sr^2+^-induced block (Figure 6—figure supplement 1b and c). The selectivity of NavPp was higher for Na^+^ than for Ca^2+^, Sr^2+^, K^+^, or Cs^+^ (Figure 6d and e; Figure 6—source data 3). The *P*_Ca_/*P*_Na_ was 0.308 ± 0.028 in a bath solution containing both Ca^2+^ and Na^+^, suggesting that a larger fraction of Ca^2+^ is allowed to permeate with outside Na^+^ ions through NavPp than through canonical BacNavs. Similar to Ca^2+^, Sr^2+^ also blocked the NavPp current, but may also permeate the channel along with Na^+^ ions (Figure 6—figure supplement 1c). These findings demonstrate a unique feature of NavPp, a low-affinity Ca^2+^ block, which is not reported in canonical BacNavs.

Interestingly, the filter sequence of NavPp, ‘TLEDWTD’, has three negatively charged residues, similar to the filter sequences of the artificial Ca^2+^-selective BacNav mutants (the ‘TLEDWSD’ mutant of NavAb and the 'TLEDWAD’ mutant of NaChBac) (Tang et al., 2014; Yue et al., 2002). NavPp does not show high Ca^2+^ permeability, but rather a Ca^2+^ block. We also investigated NavPp mutants that have the same filter sequences as the artificial Cavs. NavPp-T6S ‘TLEDWSD’ exhibited Ca^2+^-blocked currents similar to those exhibited by wild-type NavPp (Figure 6e: upper). Further, NavPp-T6A ‘TLEDWAD’ showed no inward current in bath solutions containing divalent cations, suggesting that the Ca^2+^-induced block was enforced (Figure 6e: bottom). Therefore, both of the selectivity filter sequences that provide Ca^2+^ selectivity to canonical BacNavs failed to generate Ca^2+^-permeable NavPp, indicating that the cation-permeable mechanism of NavPp differs from that of canonical BacNavs, as well as that of CavMr. On the other hand, the cells transfected with genes from *H. elongata* and *T. turnerae* failed to show any detectable currents, while these genes code selectivity filter sequences that are similar to that of NavPp (Figure 1c).

### Swapping the filter regions between CavMr and NavPp revealed the importance of the glycine residue at position 4 for Ca^2+-^selective permeation

**Figure 7.**
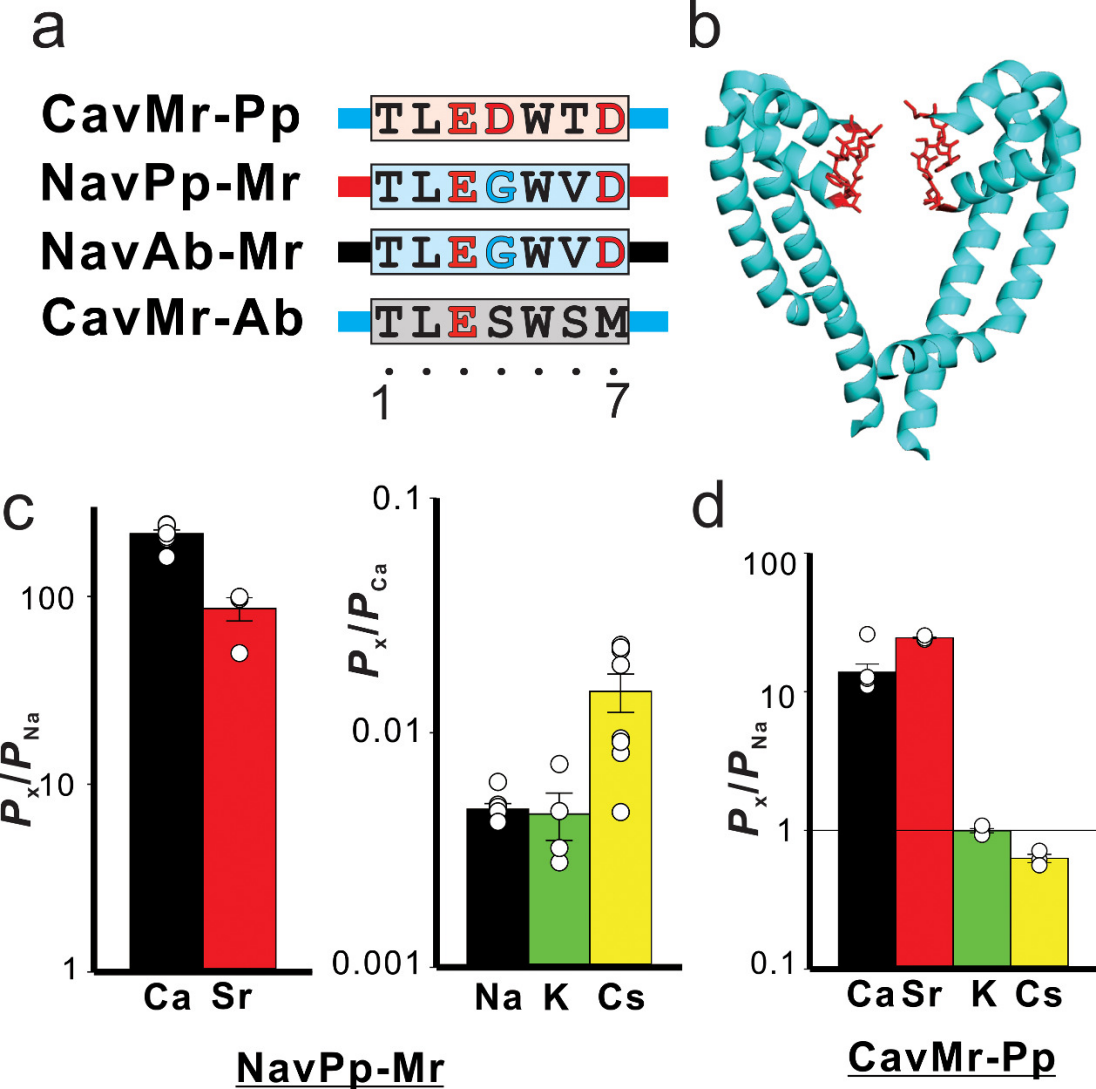
The cation selectivity of the channel mutants in which the selectivity filter is swapped between CavMr and NavPp. (**a**) Amino acid sequences of the selectivity filter in the swapped mutants, CavMr-Pp, Nav-Pp, NavAb-Mr, and CavMr-Ab. The selectivity filter sequences of CavMr, NavPp and NavAb are indicated using single-letter codes with cyan, red, and gray shade, respectively. Negatively charged residues are colored in red. Glycine residues are colored in cyan. The straight lines of cyan, red, and black indicate the other part of pore domain of CavMr, NavPp, and NavAb, respectively. (**b**) Pore domains of crystal structure of NavAb (PDB code:5YUA). The selectivity filter, which corresponds to the sequences shown in panel (a), was indicated in red. (**c**) The relative permeability of divalent cations to Na^+^ (left) and that of monovalent cations to Ca^2+^ (right) in NavPp-Mr (Figure 7—source data 1). (**d**) The relative permeability of different cation species to Na^+^ in CavMr-Pp (Figure 7—source data 2). Figure 7—source data 1.The values of relative permeability. Figure 7—source data 2.The values of relative permeability.

To search for the determinants of Ca^2+^ selectivity in CavMr, we investigated a series of mutants in which the filter regions were swapped between CavMr and NavPp (Figure 7a and b), which exhibited channel activity (Figure 7—figure supplements 1–2). A NavPp mutant whose selectivity filter was replaced with that of CavMr, named NavPp-Mr, exhibited much higher Ca^2+^ selectivity (*P*_Ca_/*P*_Na_ = 215 ± 33) as well as high Sr^2+^ selectivity, comparable to that of CavMr (Figure 7c; Figure 7—source data 1). In addition, NavPp-Mr excluded Na^+^ and K^+^ similar to CavMr, but weakly allowed Cs^+^ permeation in contrast to CavMr. On the other hand, a CavMr mutant whose selectivity filter was replaced with that of NavPp (CavMr-Pp) almost lost its Ca^2+^ selectivity (*P*_Ca_/*P*_Na_ = 13.8 ± 2.0), and was less able to discriminate Cs^+^ and K^+^ from Na^+^ (Figure 7d; Figure 7—source data 2). That is, CavMr-Pp was a more non-selective channel than the wild-type CavMr, rather than a Na^+^-selective channel. The Ca^2+^ selectivity (from NavPp to CavMr) was almost transferable, but the Na^+^ selectivity was not. We also investigated the full swapping of the filter sequences between CavMr and NavAb (Figure 7a), but neither swapped mutants of CavMr nor NavAb had detectable currents. This finding suggested that CavMr and NavAb achieve cation selectivity using different structural backbones and mechanisms.

**Figure 7—figure supplement 1.**
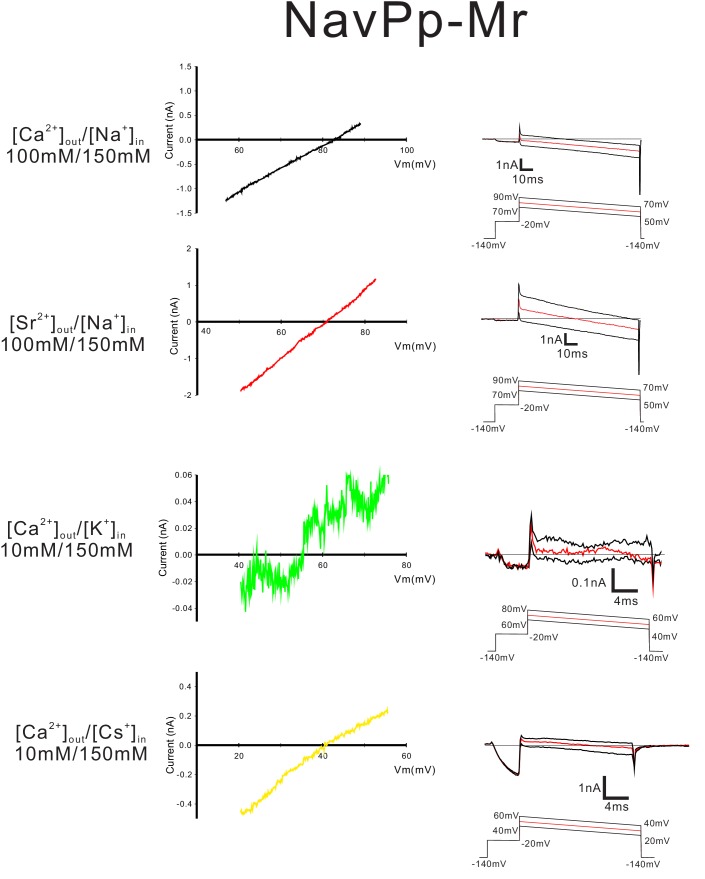
Current-voltage relationship plots and representative current traces of the ramp pulse of the CavMr-Pp selectivity-filter-swapped mutants. Current-voltage relationship plots for CavMr-Pp using the averaged currents generated with three different ramp pulses in the solutions indicated on the left side. For the evaluation of the permeability of Ca^2+^, Sr^2+^, K^+^ and Cs^+ ^relative to Na^+^, Ca^2+^ solution [100 mM CaCl_2_, 10 mM HEPES (pH 7.4 adjusted with Ca(OH)_2_) and 10 mM glucose], Sr^2+^ solution [100 mM SrCl_2_, 10 mM HEPES (pH 7.4 adjusted by Sr(OH)_2_) and 10 mM glucose], K^+^ solution [150 mM KCl, 2 mM CaCl_2_, 10 mM HEPES (pH 7.4 adjusted by KOH) and 10 mM glucose] and Cs solution [150 mM CsCl, 2 mM CaCl_2_, 10 mM HEPES (pH 7.4 adjusted by CsOH) and 10 mM glucose] were used as bath solution, respectively. High-Na^+^ pipette solution [115 mM NaF, 35 mM NaCl, 10 mM EGTA and 10 mM HEPES (pH 7.4 adjusted by NaOH)] was used for the measurements of the relative permeability of Ca^2+^ and Sr^2+^. The permeability of K^+^ and Cs^+ ^relative to Ca^2+^ was evaluated as an alternative to their permeability relative to Na^+^, because of the high Ca^2+^ selectivity of NavPp-Mr. For the evaluation, high-K^+^ pipette solution [115 mM KF, 35 mM KCl, 10 mM EGTA and 10 mM HEPES (pH 7.4 adjusted by KOH)] and high-Cs^+^ pipette solution [115 mM CsF, 35 mM CsCl, 10 mM EGTA and 10 mM HEPES (pH 7.4 adjusted by CsOH)] were used. As bath solution, Ca^2+^ solution [100 mM CaCl_2_, 10 mM HEPES (pH 7.4 adjusted with Ca(OH)_2_) and 10 mM glucose] was used. Currents were generated by the step pulse of −20 mV from −140 mV holding potential, followed by ramp pulses with different voltage values. The time courses of the change in membrane potentials are shown at the bottom of the respective current traces.

**Figure 7—figure supplement 2.**
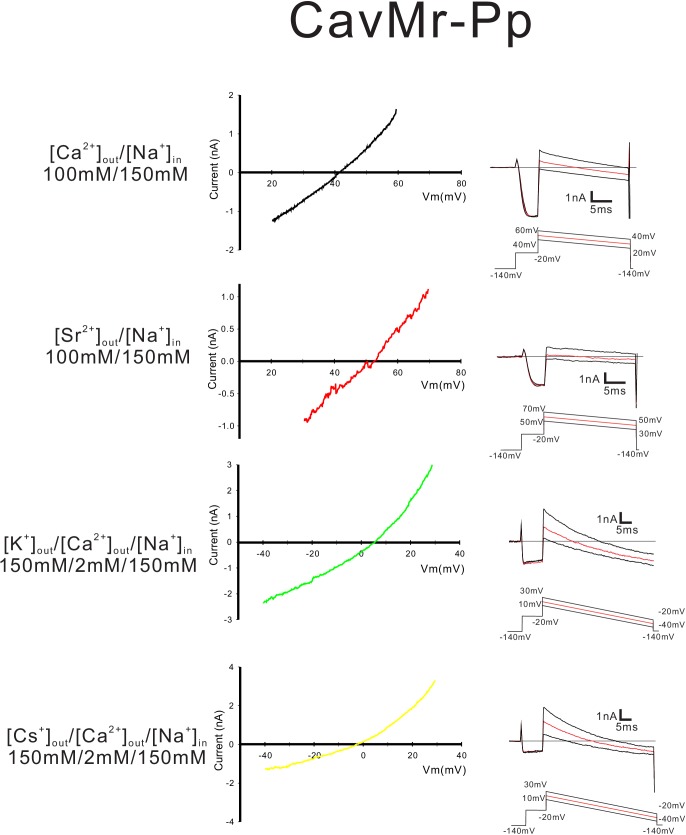
Current-voltage relationship plots and representative current traces for the ramp pulse of NavPp-Mr selectivity-filter-swapped mutants. Current-voltage relationship plots of NavPp-Mr using the averaged currents generated with three different ramp pulses in the solutions indicated on the left side. To evaluate the relative permeability of Ca^2+^, Sr^2+^, K^+^ and Cs^+^ to Na^+^, Ca^2+^ solution, Sr^2+^ solution, K^+^ solution and Cs^+^ solution were used as the bath solution, respectively. High-Na^+^ pipette solution was used as the pipette solution. The solution contents are described in the 'Materials and methods' and in Figure 7—figure supplement 1. The time courses of the membrane potentials are shown at the bottom of each trace.

**Figure 8.**
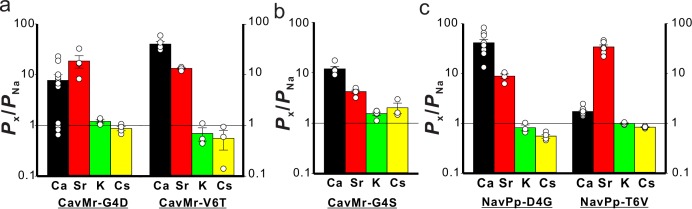
The single-point mutations that cause loss and acquistion of Ca^2+^ selectivity in CavMr and NavPp, respectively. (**a**) The permeability of each cation species relative to Na^+ ^permeability in the single-point mutants of CavMr. The selectivity filter of CavMr was changed to the corresponding residues of NavPp at position 4 (G4D) or position 6 (V6T) (Figure 8—source data 1). (**b**) The permeability of each cation species relative to Na^+ ^permeability in the G4S mutant of CavMr, whose position 4 residue of the selectivity filter was mutated to the corresponding residue of canonical BacNavs (Figure 7—source data 1). (**c**) The permeability of each cation species relative to Na^+ ^permeability in the single-point mutants of NavPp. The selectivity filter of NavPp was changed by swapping in the corresponding residues of CavMr at position 4 (D4G) or position 6 (T6V) (Figure 8—source data 3). Figure 8—source data 1.The values of relative permeability. Figure 8—source data 2.The values of relative permeability. Figure 8—source data 3.The values of relative permeability.

Positions 4 and/or 6 of the filter sequences are thought to be important for Ca^2+^-selective permeation through NavPp-Mr and CavMr, because only these two positions were mutated in the swapping experiments. We investigated which of the mutations in positions 4 and 6 had greater effects on the loss and acquisition of Ca^2+^ selectivity in CavMr and NavPp, respectively. In CavMr, two single mutants, CavMr-G4D and CavMr-V6T, both decreased Ca^2+^ selectivity and allowed K^+^ and Cs^+^ permeation (Figure 8a;Table 1; Figure 8—figure supplements 1–2; Figure 8—source data 1). The mutational effect was greater in CavMr-G4D, whose *P*_Ca_/*P*_Na_ was less than 10 (7.73 ± 2.24). CavMr-G4S, in which Gly4 was replaced with the Ser4 of NavAb, also exhibited lower Ca^2+^ selectivity (*P*_Ca_/*P*_Na_ = 11.9 ± 1.5) and was also K^+^ and Cs^+^ permeable, indicating that a minor substitution by serine allowed the channel to retain a little calcium selectivity, but the monovalent cation selectivity had completely disappeared (Figure 8b; Table 1; Figure 8—figure supplement 3; Figure 8—source data 2). In the case of NavPp, NavPp-D4G acquired divalent cation, Ca^2+^ and Sr^2+^, selectivity over Na^+^, and also showed a greater *P*_K_/*P*_Na_ and *P*_Cs_/*P*_Na_ than wild-type NavPp (Figure 8c; Table 1; Figure 8—figure supplements 4–5; Figure 8—source data 3). By contrast, NavPp-T6V failed to acquire the high Ca^2+^ selectivity (*P*_Ca_/*P*_Na_ = 1.72 ± 1.09) and also allowed K^+^ and Cs^+^ permeation, while it had relatively high Sr^2+^ selectivity. These results indicate that, in both CavMr and NavPp, a glycine residue at position 4 is a key determinant for Ca^2+^ selectivity. It is noteworthy that the glycine is a conserved residue at position 4 of subdomains I and III in all subtypes of mammalian Cavs (Figure 1b).

**Figure 8—figure supplement 1.**
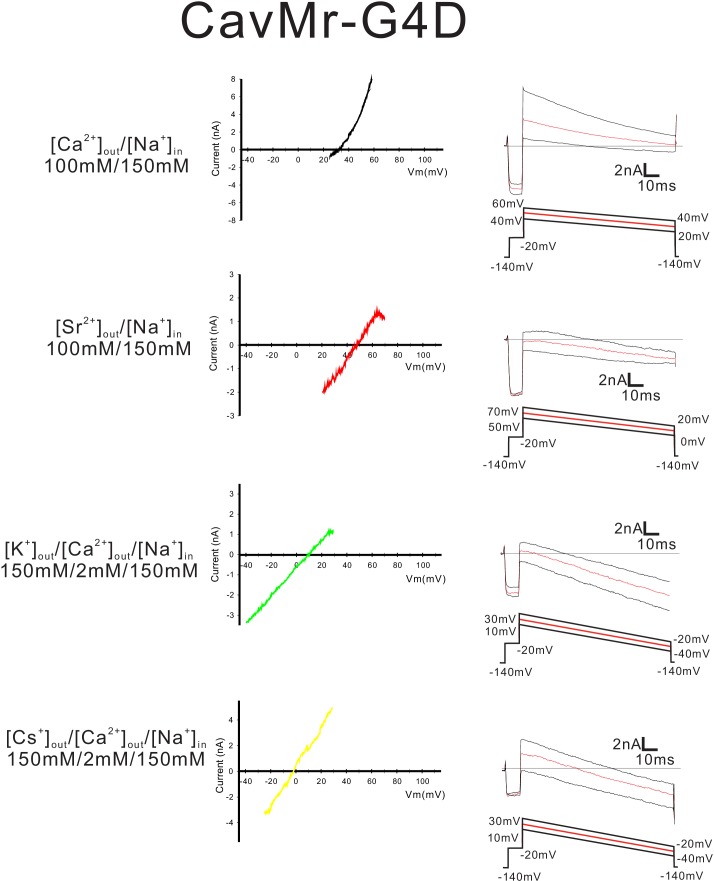
Current-voltage relationship plots and representative current traces for the ramp pulse of CavMr-G4D selectivity-filter-swapped mutants. Current-voltage relationship plots of CavMr-G4D using the averaged currents generated with three different ramp pulses in the solutions indicated on the left side. To evaluate the permeability of Ca^2+^, Sr^2+^, K^+^ and Cs^+ ^relative to that of Na^+^, Ca^2+^ solution, Sr^2+^ solution, K^+^ solution and Cs^+^ solution, respectively, was used as the bath solution. High-Na^+^ pipette solution was used as the pipette solution. The solution contents were described in the 'Materials and methods' and in Figure 7—figure supplement 1. The time courses of the changes in membrane potentials are shown at the bottom of the current traces.

**Figure 8—figure supplement 2.**
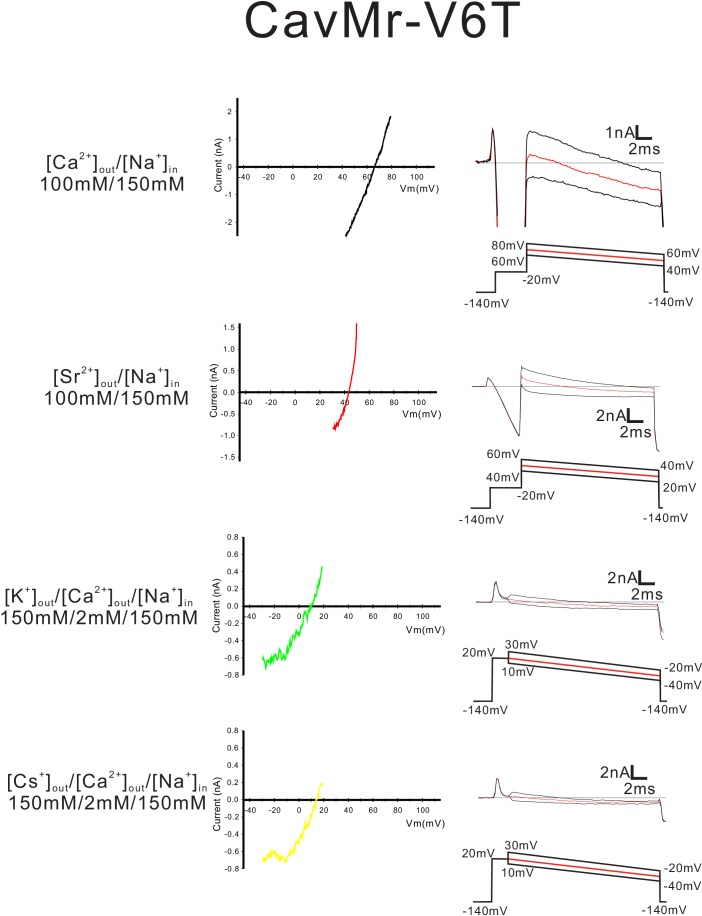
Current-voltage relationship plots and representative current traces for the ramp pulse of CavMr-V6T selectivity-filter-swapped mutants. Current-voltage relationship plots for CavMr-V6T using the averaged currents generated with three different ramp pulses in the solutions indicated on the left side. To evaluate the permeability of Ca^2+^, Sr^2+^, K^+^ and Cs^+ ^relative to that of Na^+^, Ca^2+^ solution, Sr^2+^ solution, K^+^ solution and Cs^+^ solution, respectively, were used as the bath solution. High-Na^+^ pipette solution was used as the pipette solution. The solution contents are described in the 'Materials and methods' and in Figure 7—figure supplement 1. The time courses of the changes in membrane potentials are shown at the bottom of the current traces.

**Figure 8—figure supplement 3.**
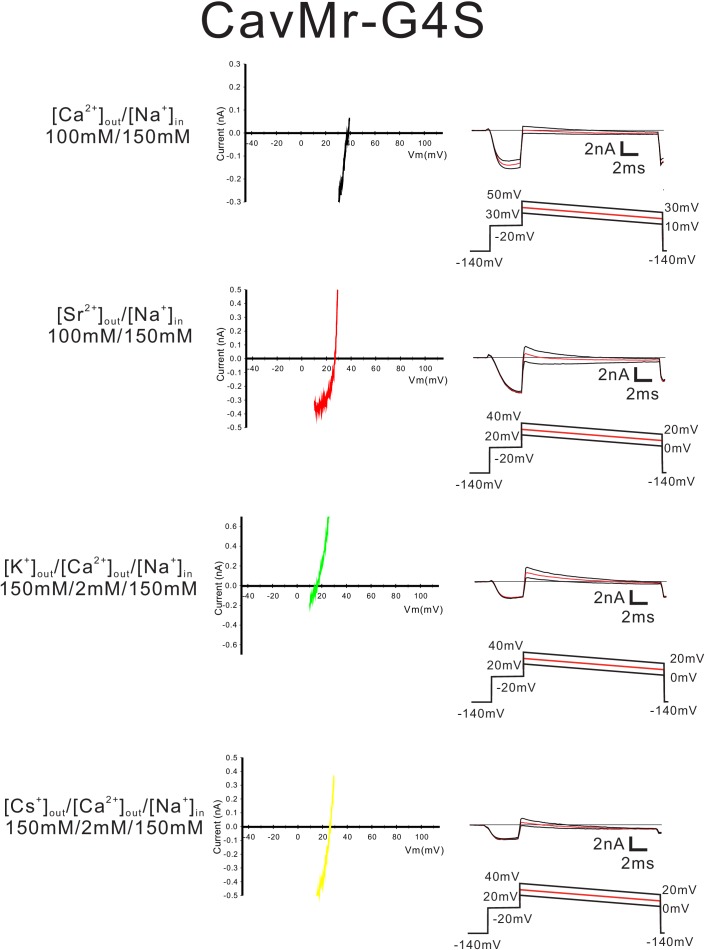
Current-voltage relationship plots and representative current traces for the ramp pulse of CavMr-G4S selectivity-filter-swapped mutants. Current-voltage relationship plots for CavMr-G4S using the currents generated with the ramp pulse (red line) in the solutions indicated on the left side. To evaluate the permeability of Ca^2+^, Sr^2+^, K^+^ and Cs^+ ^relative to that of Na^+^, Ca^2+^ solution, Sr^2+^ solution, K^+^ solution and Cs^+^ solution, respectively, were used as the bath solution. High-Na^+^ pipette solution was used as the pipette solution. The solution contents were described in the 'Materials and methods' and in Figure 7—figure supplement 1. The time courses of the changes in membrane potential are shown at the bottom of the current traces.

**Figure 8—figure supplement 4.**
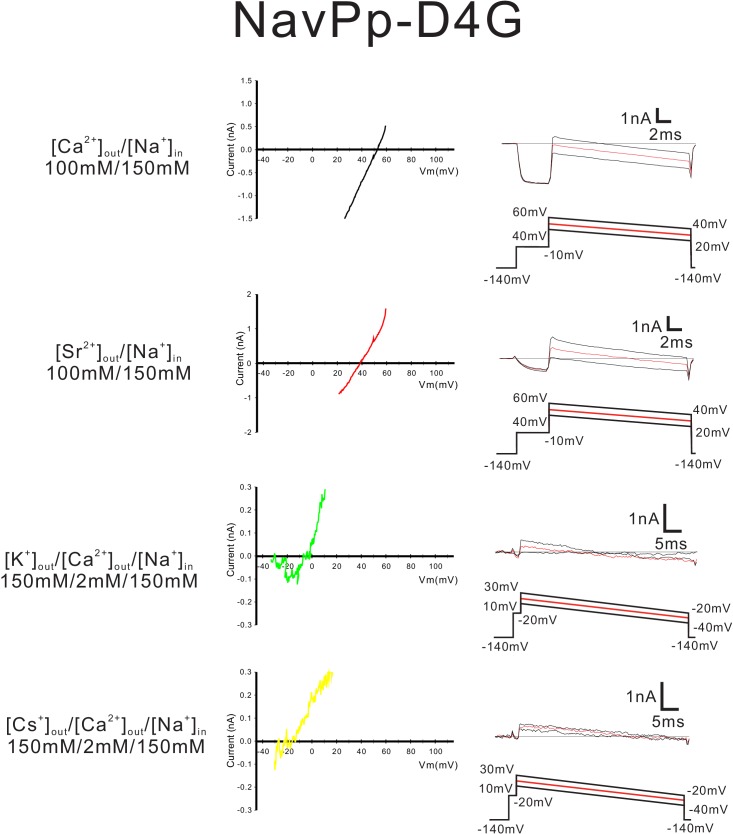
Current-voltage relationship plots and representative current traces for the ramp pulse of NavPp-D4G selectivity-filter-swapped mutants. Current-voltage relationship plots for NavPp-D4G using the averaged currents generated with three different ramp pulses in the solutions indicated on the left side. To evaluate the permeability of Ca^2+^, Sr^2+^, K^+^ and Cs^+ ^relative to that of Na^+^, Ca^2+^ solution, Sr^2+^ solution, K^+^ solution and Cs^+^ solution, respectively, were used as the bath solution. High-Na^+^ pipette solution was used. The solution contents are described in the 'Materials and methods' and in Figure 7—figure supplement 1. The time courses of the change in membrane potentials are shown at the bottom of the current traces.

**Figure 8—figure supplement 5.**
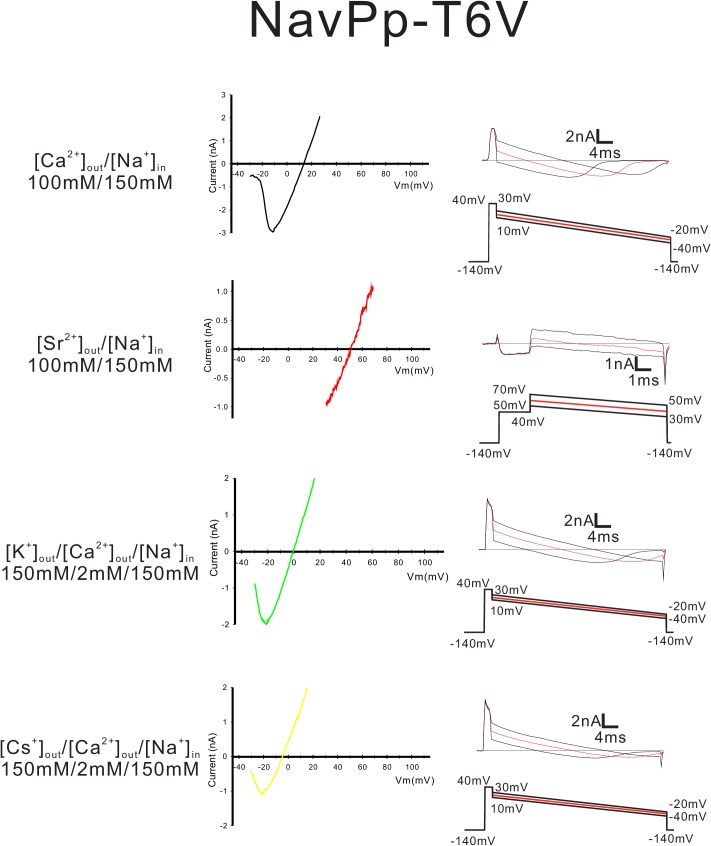
Current-voltage relationship plots and representative current traces for the ramp pulse of NavPp-T6V selectivity-filter-swapped mutants. Current-voltage relationship plots of NavPp-T6V using the averaged currents generated with three different ramp pulses in the solutions indicated on the left side. To evaluate the permeability of Ca^2+^, Sr^2+^, K^+^ and Cs^+ ^relative to that of Na^+^, Ca^2+^ solution, Sr^2+^ solution, K^+^ solution and Cs^+^ solution, respectively, were used as the bath solution. High-Na^+^ pipette solution was used. The solution contents are described in the 'Materials and methods' and in Figure 7—figure supplement 1. The time courses of the changes in membrane potentials are shown at the bottom of the current traces.

## Discussion

### A native prokaryotic voltage-dependent Ca^2+^ channel has a unique Ca^2+-^selective mechanism

In this study, we newly characterized two prokaryotic voltage-dependent cation channels, CavMr and NavPp. CavMr is the first native prokaryotic Cavs reported, and NavPp could be inhibited by high concentrations of extracellular Ca^2+^. The *P*_Ca_/*P*_Na_ of CavMr was more than 200 (Figure 3e and Table 1), comparable to that of CavAb, an artificial Ca^2+^ channel. Anomalous mole fraction effects were not observed in CavMr (Figure 4a and b), suggesting that CavMr has a very high affinity for Ca^2+^. In addition to providing new insights about general Ca^2+^-selective mechanisms, CavMr has the potential to be a new genetic tool for upregulating calcium signaling, as BacNavs are useful genetic tools for increasing action potential firing in mice (Bando et al., 2016; Kamiya et al., 2019; Lin et al., 2010).

**Figure 9.**
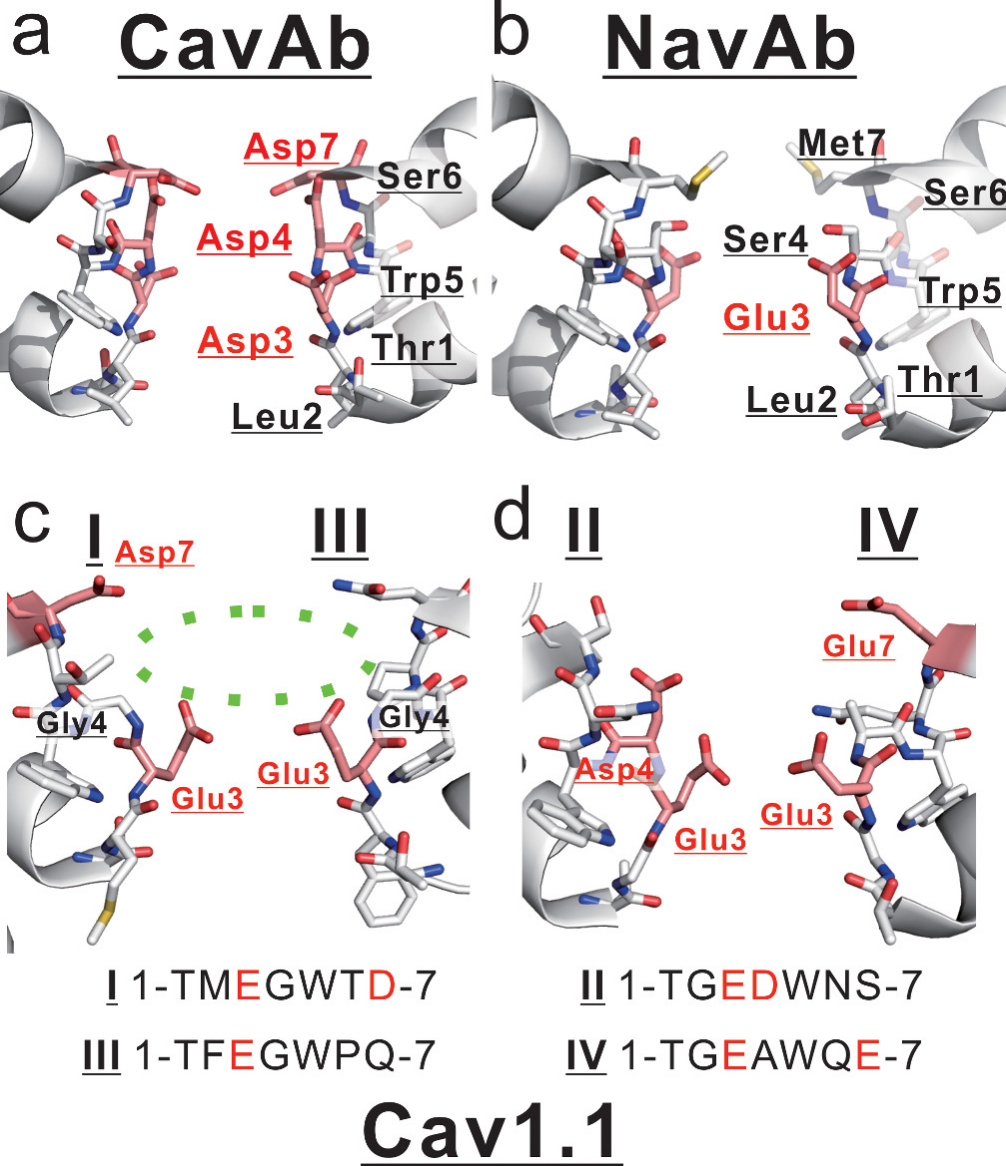
Comparison between mammalian and prokaryotic Cav. (**a, b**) Structures of the selectivity filter in CavAb (PDB code: 4MVZ) and NavAb (PDB code: 5YUA). (**c, d**) Structure of the rabbit Cav1.1 selectivity filter (PDB code: 5GJV). The subdomains I and III (**c**), and II and IV (**d**) are shown separately. The carbon atoms of negatively charged residues are indicated in pink. A dashed green circle indicates the wide entrance of the selectivity filter.

Phylogenetic analysis demonstrated that CavMr and NavPp are similar to each other, but distant from canonical BacNavs (Figure 1b). The high Ca^2+^ selectivity of CavMr was transferable to NavPp. Intriguingly, two pairs of mutants with the same selectivity filter (CavMr-G4D and NavPp-T6V, CavMr-V6T and NavPp-D4G) showed a very similar tendency with regard to both the order and extent of cation selectivity (Figure 8a and c). Therefore, the basic overall architecture of the NavPp selectivity filter could be similar to that of CavMr. On the other hand, the Ca^2+-^selectivity mechanism of CavMr completely differs from that of CavAb. Structural comparison of NavAb and CavAb showed that the aspartate mutations did not alter the main chain trace, and simply introduced the negative charges around the ion pathway to increase Ca^2+^ permeability (Figure 9a and b) (Tang et al., 2014). By contrast, in the case of CavMr, two non-charged residues (Gly4 and Val6) are required for the high Ca^2+^ selectivity (Figures 7d and 8a), whereas Asp7 is not necessary (Figure 4g). A no-charge mutation at position 7, CavMr-D7M ‘TLEGWVM’, is an outstanding example demonstrating that high Ca^2+^ selectivity can be achieved in the absence of any aspartates in its filter region. Furthermore, the introduction of a negative charge into the selectivity filter (G4D mutation) had an effect on the Ca^2+^ selectivity of CavMr that was opposite to the effect seen in NavAb and NaChBac (Tang et al., 2014; Yue et al., 2002). Moreover, the decreased selectivity of monovalent and divalent cations in G4S also indicates that the glycine at position 4 plays a crucial role in Ca^2+^ selectivity in CavMr (Figure 8b). The flexibility and/or small size of the glycine at position 4 in CavMr might be critical.

These findings are inconsistent with those derived from the Ca^2+^-selective mutants of NavAb and NaChBac, and therefore the native structure of the selectivity filter and the molecular mechanism of ion selectivity of CavMr are thought to differ from those of CavAb. The structure of CavMr is not yet available, but we are able to speculate on the structure of the selectivity filter of CavMr, based on the structure of human Cav1.1 subdomains I and III (Wu et al., 2016) (Figure 9c), whose selectivity filter sequences are very similar to that of CavMr. In the selectivity filter of Cav1.1 subdomains I and III, the side chain of the residue at position 7 is shifted outward. The position 4 glycine residue widens the entrance of the selectivity filter, facilitating the entry of hydrated cations into the ion pore and possibly increasing Ca^2+^ selectivity.

### The roles of Cavs in prokaryotes and the species-specific tuning of homo-tetrameric channels

Prokaryotes have a number of putative Ca^2+^-binding proteins, such as EF-hand proteins, P-type Ca^2+^ pumps, and Ca^2+^ transporters (Domínguez et al., 2015). The intracellular Ca^2+^ concentration is kept low and changes in response to mechanical and chemical stimuli (Dominguez, 2004). These features imply that prokaryotic Ca^2+^ signaling is similar to that of eukaryotes. The strong ability of CavMr to exclude Na^+^ and K^+^ along with Ca^2+^ permeation suggests that its primary physiological role is Ca^2+^ intake in response to a voltage change (Figure 3e and f). In some bacteria, the direction of flagellar rotation and chemotaxis changes depending on the internal Ca^2+^ concentration (Ordal, 1977; Tisa et al., 1993; Tisa and Adler, 1995). *M. ruber* was isolated from hot springs, and therefore a sufficient amount of Ca^2+^ is likely to exist in its native environment (Loginova et al., 1984). CavMr activation by a voltage change, which could vary depending on the environmental ionic conditions, might lead to any response that allows adaptation to the new environment, such as flagellar rotation. These characteristics indicate the existence of signal coupling between the membrane voltage and Ca^2+^, even in the early stages of life, which might be the origin of the corresponding functions in eukaryotes, such as muscle contraction.

NavPp permeates more Na^+^ than Ca^2+^, but its selectivity is modest (Figure 6d and Table 1). Notably, *P. pacifica* is a marine myxobacterium that requires NaCl for its growth (Iizuka et al., 2003). As mentioned above, the basic architecture of the CavMr/NavPp group is thought to produce a preference for Ca^2+^. *P. pacifica* might modify this channel architecture to acquire a Na^+^ intake pathway, which would probably result in the remaining feature of low-affinity Ca^2+^ inhibition in NavPp. This flexible usage of homo-tetrameric channels to allow different cations to permeate is also reported in another bacterium, *Bacillus alkalophilus* (DeCaen et al., 2014). NsvBa from *B. alkalophilus* is a non-selective channel whose selectivity filter is changed from ‘TLESWAS’, a typical Na^+^-selective sequence in alkaliphilic bacillus, to ‘TLDSWGS’, possibly as an adaptation to its ionic environment. Recently, an early eukaryote, diatom, was found to have another homo-tetrameric channel with no selectivity, namely EukCat, which has an important role in electrical signaling in this species (Helliwell et al., 2019). These findings suggest that the cation selectivity of the homo-tetrameric channel family can be flexibly tuned to realize the required roles specific to its original species.

### Insights into Ca^2+^ selectivity and the evolution of Cavs

Aspartate residues are generally observed in the Ca^2+^ permeation pathway in ion channels, as well as in many Ca^2+^-binding proteins (Halling et al., 2016; Yan et al., 2015; Zalk et al., 2015). Actually, NavAb and NaChBac were successfully transformed into Ca^2+^-selective channels with the aspartate-introduced filter sequences ‘TLDDW(S/A)D’ (Tang et al., 2014; Yue et al., 2002). But, our results elucidate that this strategy is not the only way to achieve high Ca^2+^ selectivity. Human Cav subdomains possess, at most, two aspartate residues in their selectivity filters in a part other than position 3. In addition, the negatively charged residue at position 3, which is thought to be the most critical for cation selectivity in both Navs and Cavs, is not aspartate but glutamate in most of the human Cav subdomains (Yu and Catterall, 2004). CavAb has 12 aspartates in the selectivity filter of its channel tetramer, while there are four aspartates in CavMr. The net negative charge is 5~7 in mammalian Cavs, 8 in CavMr, and 12 in CavAb. As shown in mammalian Cavs, Ca^2+^ selectivity can be achieved with even fewer negative charges in the selectivity filter than is the case in CavAb, which suggest that the calcium-selective mechanism requires a specific backbone structure of the pore domain depending on its selectivity filter charges.

The members of the AnclNav group can be found in a variety of bacterial phyla and in archaea (Figure 1—figure supplement 1). In particular, the *Deinococcus*-*Thermus* phylum, in which *M. ruber* is included, is considered to be relatively close to the universal ancestor of life (Hug et al., 2016). It is also notable that the filter sequence of NavPp is completely the same as that of the ancestral BacNav predicted in the previous report [TLED(or S in equal probability)WTD] (Liebeskind et al., 2013). These pieces of evidence suggest that the AnclNav group preserves the feature of an ancestral BacNav.

In our comprehensive phylogenetic analysis, all of the one-domain type of channel groups, including the AnclNav group, are almost equally distant from the root of the divided subdomains of eukaryotic 24TM Cavs/Navs (Figure 1—figure supplement 2). Again, this information is insufficient to allow us to deduce any conclusion for a eukaryotic ancestor of 24TM channels just before subdomain duplication. It is noteworthy, however, that the selectivity filter sequence of CavMr is very similar to those of human Cav subdomains I and III, both of which possess a glycine at position 4 (Figure 9c). In particular, the Cav3.1 and 3.2 subdomains I have the same sequence as the evolutionally distant CavMr. These sequence similarities of the glycine residue at position 4 are also found in CatSper, the sperm calcium permeable channels (Darszon et al., 2011), which branches close from the convergent point of four subdomains. The channel region of CatSper is formed by four different subunits (CatSper1–4). The selectivity filters of CatSper 3 ‘TVDGWTD’ and CatSper 4 ‘TQDGWVD’ are similar to that of CavMr. Taken together, these findings suggest that the selectivity filter of eukaryotic ancestor of 24TM channels might have been similar to those of AnclNavs, especially to that of CavMr.

In the future, information about the structure of these homo-tetrameric channels could help us to gain a deeper understanding of channel evolution, and further investigation of the detailed structure of CavMr may help us to elucidate the principles and origin underlying Ca^2+^ selectivity.

## Materials and methods

### Cloning of BacNav homologs and site-directed mutagenesis

The NaChBac amino acid sequence (NP_242367) was used as the query for a BLASTP search against the Microbial Genomic database at NCBI. The identified primary sequence data were obtained from Entrez at NCBI (*Meiothermus ruber* as ZP_04038264, *Plesiocystis pacifica* as ZP_01909854, *Halomonas elongata* as YP_003896792 and *Teredinibacter turnerae* as YP_003073405). These DNAs were synthesized by Genscript Inc and subcloned into the pCI vector (Promega) using the EcoRI and SalI sites and the pBiEX vector (Novagen) using the NcoI and BamHI sites, respectively. Site-directed mutagenesis was achieved by polymerase chain reaction (PCR) of the full-length plasmid containing the Nav gene using PrimeSTARMAX DNA Polymerase (Takara Bio.). All clones were confirmed by DNA sequencing.

### Phylogenetic analysis

Phylogenetic analyses were performed using the Molecular Evolutionary Genetics Analysis (MEGA X) software (Kumar et al., 2016). Protein sequences of putative BacNavs were collected by reference to a previous report (Liebeskind et al., 2013) and using multi-round searches of the NCBI database using NaChBac, NavAb, CavMr and NavPp as templates. Multiple sequence alignment was generated with MUSCLE contained in MEGA X. To generate the phylogenetic tree of BacNavs, the targeted sequences to be analyzed were selected using GBLOCKS0.91B (Castresana, 2000). The least stringent parameters selected 167 amino acids that cover most of transmembrane domains and the S4/S5 linker. For comparison with the eukaryotic Navs, Cavs, CatSper and EukCatA, the sequences of these proteins were collected by reference to the previous studies of Nav/Cav evolution (Cai and Clapham, 2008; Gur Barzilai et al., 2012; Helliwell et al., 2019; Liebeskind et al., 2011). The sequences of four-domain type of Navs and Cavs were divided to each subdomain. These divided subdomain sequences were aligned with EukCatA, CatSper and BacNavs using MUSCLE, and then the targeted sequences, which correspond to the selected amino acids in the analysis of BacNavs, were extracted. Regions that are poorly conserved between BacNavs and eukaryotic channels were manually removed, leaving 162 amino acids. Maximum likelihood trees were generated using MEGA X. The model validation was performed and determined to be a LG+G+F model in both the analyses, with and without eukaryotic channels. The sequence logos that indicate the frequencies at which different amino acids appear in the selectivity filter were generated with WebLogo (Crooks et al., 2004).

### Electrophysiological analysis using mammalian cells

For the recordings related to mole fraction effects (Figure 4a–d), currents were recorded from Chinese hamster ovary (CHO)-K1 cells (ATCC catalog number CCL-61) that expressed the channels. The recordings were performed as described previously (Tateyama and Kubo, 2018). Cells were transfected with channel DNAs using the LipofectAMINE 2000 (Invitrogen) and plated onto cover slips. Currents were recorded 24–36 hr after transfection. Current recording by the whole-cell patch-clamp technique was performed using Axopatch 200B amplifiers, Digidata1332A, and pClamp nine software (Molecular Devices). The pipette solution contained 130 mM KCl, 5 mM Na_2_-ATP, 3 mM EGTA, 0.1 mM CaCl_2_, 4 mM MgCl_2_ and 10 mM HEPES (pH 7.2 adjusted with KOH). The bath solution contained 135 mM NaCl, 4 mM KCl, 1 mM CaCl_2_, 5 mM MgCl_2_ and 10 mM HEPES (pH 7.4 adjusted with NaOH). For the measurement of mole fraction effects, bath solutions containing different ratios of NaCl/CaCl_2_ (135/0, 108/18, 81/36, 54/54, 27/82 and 0/90 mM) were used. A Ca^2+^-free solution was achieved by using a solution containing 135 mM NaCl, 1 mM EGTA and 0 mM CaCl_2_.

### Electrophysiological measurement in insect cells

Recordings other than those for mole fraction effects were performed using SF-9 cells. SF-9 cells (ATCC catalog number CRL-1711) were grown in Sf-900 III medium (Gibco) complemented with 0.5% 100 × antibiotic antimycotic (Gibco) at 27°C. Cells were transfected with target channel-cloned pBiEX vectors and enhanced green fluorescent protein (EGFP)-cloned pBiEX vectors using Fugene HD transfection reagent (Promega). The channel-cloned vector (2 μg) was mixed with 0.5 μg of the EGFP-cloned vector in 100 μL of the culture medium. Next, 3 μL Fugene HD reagent was added and the mixture was incubated for 10 min before the transfection mixture was gently dropped onto cultured cells. After incubation for 16–48 hr, the cells were used for electrophysiological measurements. In the measurement of I–V relation curves, the pipette solution contains 75 mM NaF, 40 mM CsF, 35 mM CsCl, 10 mM EGTA, and 10 mM HEPES (pH 7.4 adjusted by CsOH).

For evaluation of ion selectivity, a high-Na^+^ pipette solution [115 mM NaF, 35 mM NaCl, 10 mM EGTA, and 10 mM HEPES (pH 7.4 adjusted by NaOH)] was used. For the evaluation of Ca^2+^, Sr^+^, K^+^ and Cs^+^ selectivity, Ca^2+^ solution [100 mM CaCl_2_, 10 mM HEPES (pH 7.4 adjusted by Ca(OH)_2_), and 10 mM glucose], Sr^2+^ solution [100 mM SrCl_2_, 10 mM HEPES (pH 7.4 adjusted by Sr(OH)_2_), and 10 mM glucose], K^+^ solution [150 mM KCl, 2 mM CaCl_2_, 10 mM HEPES (pH 7.4 adjusted by KOH), and 10 mM glucose], and Cs^+^ solution [150 mM CsCl, 2 mM CaCl_2_, 10 mM HEPES (pH 7.4 adjusted by CsOH), and 10 mM glucose], respectively, were used as the bath solution. *E*_rev_ of highly Ca^2+-^selective channels were measured under three external solutions containing: 144 mM NMDG-Cl and 4 mM CaCl_2_; 135 mM NMDG-Cl and 10 mM CaCl_2_; and 120 mM NMDG-Cl and 20 mM CaCl_2_ (10 mM HEPES pH 7.4 adjusted with HCl). *E*_rev_ of highly Ca^2+-^selective channels for the calculation of *P*_K_/*P*_Ca_ and *P*_Cs_/*P*_Ca_ were measured under external solutions containing 135 mM NMDG-Cl and 10 mM CaCl_2_ (10 mM HEPES pH 7.4 adjusted with HCl) with high-K^+^ pipette solution [115 mM KF, 35 mM KCl, 10 mM EGTA, and 10 mM HEPES (pH 7.4 adjusted by KOH)] and high-Cs^+^ pipette solution [115 mM CsF, 35 mM CsCl, 10 mM EGTA, and 10 mM HEPES (pH 7.4 adjusted by CsOH)], respectively. *E*_rev_ of NavPp for the calculation of *P*_Ca_/*P*_Na_ or *P*_Sr_/*P*_Na_ were measured in an external solution containing 50 mM NMDG-Cl, 40 mM NaCl, 40 mM CaCl_2_ or SrCl_2_ and 10 mM HEPES (pH 7.4) adjusted with NaOH. *E*_rev_ of NavPp for the calculation of *P*_Cs_/*P*_Na_ was measured using a high-Cs^+^ pipette solution and an external solution containing 110 mM NMDG-Cl, 40 mM NaCl, 3 mM CaCl_2_ and 10 mM HEPES (pH 7.4) adjusted with NaOH.

As the pipette solution for measurement of the Ca^2+^ block effect in NavPp, low-Na^+^ pipette solution [140 mM CsF, 10 mM NaCl, 10 mM EGTA, and 10 mM HEPES (pH 7.4 adjusted by CsOH)] and high-Na^+^ pipette solution were used for inward and outward current measurement, respectively. As a bath solution, Ca^2+^ blocking solution [30 mM NaCl, 120 mM NMDG-Cl, 1.5 mM CaCl_2_, 10 mM HEPES (pH 7.4 adjusted by NaOH) and 10 mM glucose] was used for the 1.5 mM Ca^2+^ blocking condition. In 10 mM Ca^2+^ blocking condition, a bath solution contains 30 mM NaCl, 105 mM NMDG-Cl, 10 mM CaCl_2_, 10 mM HEPES (pH 7.4 adjusted by NaOH) and 10 mM glucose. In each Ca^2+^ blocking condition, 15 mM NMDG-Cl was replaced by 10 mM CaCl_2_. Cancelation of the capacitance transients and leak subtraction was performed using a programmed P/10 protocol delivered at −140 mV. The bath solution was changed using the Dynaflow Resolve system. All experiments were conducted at 25 ± 2°C. Cells that have a leak current smaller than 0.5nA were used for measurement. When any outliers were encountered, these outliers were excluded if any abnormalities were found in other measurement environments, but were included if no abnormalities were found. All results are presented as mean ± standard error.

### Calculation of ion selectivity by the GHK equation

To determine the ion selectivity of each channel, the intracellular solution and extracellular solution were arbitrarily set and the reversal potential at each concentration was measured by giving the ramp pulse of membrane potential. The applied ramp pulse was set to include the reversal potential. In addition, a depolarization stimulus of 2–10 ms was inserted to check whether the behavior of the cell changed for each measurement. As a result, *P*_Ca_/*P*_Na_ was calculated by substituting the obtained reversal potential (*E*_rev_) into the expression derived from the GHK equation (Frazier et al., 2000);

$$P_{Ca}/P_{Na}=\frac{-([{Na}^{+}{]}_{in}-[{Na}^{+}{]}_{out}e^{-E_{rev}F/RT})(1-e^{-2E_{rev}F/RT})}{4([{Ca}^{2+}{]}_{in}-[{Ca}^{2+}{]}_{out}e^{-2E_{revF/RT}})(1-e^{-E_{rev}F/RT})}$$

where *F* is Faraday’s constant, *R* is the Gas constant, and *T* is 298.1 (K). The same expression was used for Sr^2+^. The Sr^2+^ selectivity (*P*_Sr_/*P*_Na_) was measured in the same way.

Na^+^ selectivity against monovalent cations (*P*_M_/*P*_Na_) was calculated by substituting the obtained reversal potential and *P*_Ca_/*P*_Na_ into the expression derived from the GHK equation (Lopin et al., 2012):

$$P_{M}/P_{Na}=[\frac{-4([{Ca}^{2+}{]}_{in}-[{Ca}^{2+}{]}_{out}e^{-2E_{rev}F/RT})(1-e^{-E_{rev}F/RT})}{\left([{Na}^{+}{]}_{in}-[{Na}^{+}{]}_{out}e^{-E_{rev}F/RT})(1-e^{-2E_{rev}F/RT}\right)}∙(P_{Ca}/P_{Na})-1][\frac{\left([{Na}^{+}{]}_{in}-[{Na}^{+}{]}_{out}e^{-E_{rev}F/RT}\right)}{\left([M^{+}{]}_{in}-[M^{+}{]}_{out}e^{-E_{rev}F/RT}\right)}]$$

## Data Availability

Protein sequence data are available in the NCBI protein database. The following previously published datasets were used: LucasSCopelandALapidusAGlavinadel Rio TDalinETiceHBruceDGoodwinLPitluckSKyrpidesNMavromatisKIvanovaNMarkowitzVChengJ-FHugenholtzPWoykeTWuDTindalBKlenkH-P and Eisen JA2009Ion transport protein [Meiothermus ruber DSM 1279]NCBI ProteinZP_04038264 ShimketsLFerrieraSJohnsonJKravitzSBeesonKSuttonGRogersY-HFriedmanRFrazierM and Venter JC2010K+ transporter, Kef-type [Plesiocystis pacifica SIR-1]NCBI ProteinZP_01909854

## Additional files

Transparent reporting form
